# Investigating Different Forms of Hydrogen Sulfide in Cerebrospinal Fluid of Various Neurological Disorders

**DOI:** 10.3390/metabo11030152

**Published:** 2021-03-08

**Authors:** Viviana Greco, Cristina Neri, Damiana Pieragostino, Alida Spalloni, Silvia Persichilli, Matteo Gastaldi, Nicola B. Mercuri, Patrizia Longone, Andrea Urbani

**Affiliations:** 1 Department of Basic Biotechnological Sciences, Intensivological and Perioperative Clinics, Università Cattolica del Sacro Cuore, 00168 Rome, Italy; viviana.greco@unicatt.it (V.G.); silvia.persichilli@unicatt.it (S.P.); 2 Molecular and Genomic Diagnostics Unit, Fondazione Policlinico Universitario A. Gemelli IRCCS, 00168 Rome, Italy; 3 Thermo Fisher Scientific, 20090 Rodano, Italy; cristina.neri@thermofisher.com; 4 Department of Innovative Technologies in Medicine & Dentistry, University “G. D’Annunzio” of Chieti-Pescara, 66100 Chieti, Italy; dpieragostino@unich.it; 5 Center for Advanced Studies and Technology (CAST), Analytical Biochemistry and Proteomics Laboratory, University “G. D’Annunzio” of Chieti-Pescara, 66100 Chieti, Italy; 6 Molecular Neurobiology Unit, Experimental Neurology, IRCCS Fondazione Santa Lucia, 00143 Rome, Italy; a.spalloni@hsantalucia.it (A.S.); p.longone@hsantalucia.it (P.L.); 7 Laboratory of Neuroimmunology, IRCCS, National Neurological Insitute C. Mondino Foundation, 27100 Pavia, Italy; matteo.gastaldi@mondino.it; 8 Department of Experimental Neuroscience, IRCCS Fondazione Santa Lucia, 00143 Rome, Italy; mercurin@med.uniroma2.it; 9 Department of Systems Medicine, Università di Roma “Tor Vergata”, 00133 Rome, Italy

**Keywords:** cerebrospinal fluid, hydrogen sulfide, metabolomics, neurodegeneration, proteomics, S-sulfhydration

## Abstract

Over the past 30 years a considerable amount of data has accumulated on the multifaceted role of hydrogen sulfide (H_2_S) in the central nervous system. Depending on its concentrations, H_2_S has opposite actions, ranging from neuromodulator to neurotoxic. Nowadays, accurate determination of H_2_S is still an important challenge to understand its biochemistry and functions. In this perspective, this study aims to explore H_2_S levels in cerebrospinal fluid (CSF), key biofluid for neurological studies, and to assess alleged correlations with neuroinflammatory and neurodegenerative mechanisms. A validated analytical determination combining selective electrochemical detection with ion chromatography was developed to measure free and bound sulfur forms of H_2_S. A first cohort of CSF samples (*n* = 134) was analyzed from patients with inflammatory and demyelinating disorders (acute disseminated encephalomyelitis; multiple sclerosis), chronic neurodegenerative diseases (Alzheimer disease; Parkinson disease), and motor neuron disease (Amyotrophic lateral sclerosis). Given its analytical features, the chromatographic method resulted sensitive, reproducible and robust. We also explored low molecular weight-proteome linked to sulphydration by proteomics analysis on matrix-assisted laser desorption/ionization-time of flight mass spectrometry (MALDI-TOF MS). This study is a first clinical report on CSF H_2_S concentrations from neurological diseases and opens up new perspectives on the potential clinical relevance of H_2_S and its potential therapeutic application.

## 1. Introduction

In the last two decades, hydrogen sulfide (H_2_S) has emerged as the third “gasotransmitter” in addition to nitric oxide (NO) and carbon monoxide (CO), performing different functions in biological systems, both in health and disease conditions. H_2_S acts as a smooth muscle relaxant [1], modulates inflammation [2], and has cardioprotective effects [3]. Interestingly, several physiologic roles have been widely shown in the central nervous system (CNS) [4,5].

Endogenous H_2_S can be produced via non-enzymatic and enzymatic pathways. The non-enzymatic route, which is less clear and may occur by glucose, glutathione, and inorganic and organic polysulfide [6], is less essential for the synthesis of H_2_S.

At least three key enzymes are involved in H_2_S physiological synthesis in the organism. Endogenous H_2_S is mainly generated by cystathionine beta-synthase (CBS) and cystathionine gamma-lyase (CSE) from L-cysteine and homocysteine. Additionally, H_2_S can be also produced by 3-mercaptopyruvate sulfotransferase (3-MST) from 3-mercaptopyruvate, a cysteine and α-ketoglutarate metabolism product, in combination with cysteine aminotransferase (CAT).

Based on evidence in the brains of both human and mouse models, the tissue localization of these enzymes seems very heterogeneous. CBS is well recognized as the main enzyme for H_2_S synthesis in the brain and is mainly located in astrocytes [7,8] and microglial cells [9]. However, CSE, expressed primarily in cardiovascular system, is also found in microglial cells [10], in spinal cord [11], and in cerebellar granule neurons [12]. MST is present in the brain as well, although its expression seems to be limited to neurons [13].

After its enzymatic production, H_2_S can be immediately released, acting with various effects in different tissues, including the brain. In physiological saline solution (37 °C, pH 7.4), H_2_S is present for the most part (about four-fifths) as HS^−^ plus a trace of S^2−^ and only slightly less than one-fifth in its undissociated volatile form H_2_S. Once produced, H_2_S can be also stored in two forms of sulfur deposits: acid labile sulfur and bound sulfur, which are released in response to particular physiological signals. Acid-labile sulfur species are mainly stored in the iron–sulfur centers of mitochondrial enzymes, and released under acidic conditions. Conversely, bound sulfur species, located in the cytoplasm, are known to release H_2_S under reducing conditions [14]. Since their first description in 1993 as sulfur atoms contributing to HS^−^ generation by reducing agents [15], bound sulfur has gained increasing attention due to its involvement in mammalian physiological functions over the years [16,17]. In particular, bound sulfur species (BSS) are defined as sulfur atoms covalently bound to sulfur. BSSs include two groups: low molecular weight sulfur (cysteine persulfides, cysteine polysulphides, glutathione persulfides, glutathione polysulphides, hydrogen polysulphides), and high molecular weight sulfur which takes in persulfides bound to proteins and protein-bound polysulphides [17].

As Snyder et al. stated, H_2_S signaling modulates several physiological processes in the CNS via S-sulfhydration [18]. S-Sulfhydration (or Persulfidation) is a post-translational modification (PTM), similar to nitrosylation [19,20], that occurs on reactive cysteine residues in proteins resulting in the conversion of a cysteine -SH group into a -SSH or a persulfide group, and regulating the protein functions. The reaction forming the -SSH group is also referred to as “persulfidation” [21]. The mechanism of S-sulfhydration/persulfidation is not fully understood. While it is not yet clear how persulfidation can actually be caused by the binding of H_2_S to the thiol residue of cysteine, both of which have a valence number of −2 [22], on the other hand it is known that H_2_S alone can also cause persulfidation of proteins by reacting with sulfenic acid [23,24].

Although mechanisms linked to the production and release of H_2_S-derived polysulfides are not yet well understood, polysulfides are known to induce sulfhydration more efficiently than parental H_2_S itself [25]. Identified in the rat brain [26], polysulfides modulate the activity and function of many proteins through sulfhydration (sulfuration), including the activation of Parkin to protect neurons [27], or the translocation of NF-kB to the nucleus for the activation of anti-apoptotic genes [28].

H_2_S was first reported as a neuromodulator in the brain, mainly contributing to facilitate hippocampal long-term potentiation at physiological concentrations [5]. Therefore, H_2_S may also have relevance for neurologic disorders through its antioxidant, anti-inflammatory, and anti-apoptotic effects. However, the neuroprotective effect of H_2_S is concentration-dependent. Its dual function is currently emerging. It acts as a neuromodulator and neuroprotectant [29,30] at low concentrations (from nanomolar to low micromolar), while, at higher concentrations (from mid to high micromolar), H_2_S arises as a proinflamatory agent, promotes cell death (necrosis/apoptosis), inhibits mitochondrial function, and, in turn, facilitates the accumulation of reactive oxygen species (ROS) and excessive alteration in calcium homeostasis [12,31,32,33].

To date, several techniques, such as colorimetric methods [34], electrochemical assays [35], gas chromatography MS analysis [36], and the use of fluorescent probes [37,38], have been developed for the detection of H_2_S. The concentrations of free H_2_S, its metabolites, and H_2_S-bound forms have already been measured in different biological sources, such as urine, feces, blood, serum, tissues, and breath.

With regard to the brain studies, the analysis of endogenous sulfide levels in biological samples derived from animal models, in particular rat or mouse brain tissues, has broadened knowledge on the neuroprotective and neuromodulatory functions of H_2_S [5,14,39].

Similarly, inferences on the involvement of H_2_S in neurological diseases also arise from studies on animal models. Various CNS diseases, such as Alzheimer [40,41], Parkinson [42,43], Huntington [44], and Amyotrophic Lateral Sclerosis [45] are associated with perturbed H_2_S levels.

These findings open up new perspectives in the evaluation of H_2_S-based clinical treatments. In experimental models of AD, treatment with a H_2_S donor slows down the progression of the disease [46]. To date, H_2_S therapy is still in a preliminary phase of preclinical medical research.

Based on the dose-dependent double action of H_2_S, as cited above, it is noteworthy that only a relatively low dose of H_2_S could provide a beneficial and neuroprotective effect [47].

Therefore, the exact endogenous concentration of H_2_S cannot be disregarded.

The accurate determination of H_2_S concentrations in various biological materials, depending on the production site, still represents an important challenge in understanding its biochemistry and its functions.

Few studies on humans confirm perturbed H_2_S concentrations related to different neurological diseases, such as its decrease in plasma from AD patients [48], and, conversely, its increase in Down syndrome [49].

However, human investigations are still in their infancy, and mainly include basic medical research.

To the best of our knowledge, no direct clinical studies based on H_2_S measurements related to neurological disorders have been performed so far.

In addition, H_2_S concentrations have not yet been investigated in human samples of cerebrospinal fluid (CSF) from patients with neurodegenerative disorders.

CSF is a colorless body fluid that circulates inside the ventricular spaces of the brain, and in the subarachnoid spaces surrounding the brain and spinal cord. Although a consistent part of the brain parenchyma interstitial fluid is likely drained by the glymphatic system [50] and is not easy to collect, this fluid and its proteome still offer the best available picture of the brain status and a highly informative window for research of new neurological biomarkers [51,52,53].

The predictive role of CSF H_2_S has just been investigated in patients with central nervous system leukemia [54]. Conversely, in human CSF samples, the expression of the enzymes synthetizing H_2_S, has been recently evaluated [55].

An accurate determination of CSF H_2_S concentrations could be definitely very helpful in clarifying its physiologic role in the brain and its involvement in the brain-associated disorders. Moreover, it may provide useful information for drawing potential clinical treatment strategies.

Based on these evidences, the aim of this study was to explore the levels of H_2_S and its related forms (free H_2_S and bound sulfur species) in the CSF of various CNS conditions and evaluate putative differences. To begin with, we have analyzed an initial set of 137 CSFs from various neurological diseases including: inflammatory and demyelinating autoimmune disorders such as Acute Disseminated Encephalomyelitis (ADEM) and Multiple Sclerosis (MS); chronic neurodegenerative diseases such as Alzheimer’s disease (AD); degenerative disorders of the motor system as Parkinson’s disease (PD); motor neuron diseases such as Amyotrophic lateral sclerosis (ALS). Controls (CTRL) were made of CSF samples from individuals without neurological or inflammatory diseases.

To quantify both forms, free and bound sulfur (specifically high molecular weight sulfur), we have optimized the CSF sample preparation protocol and developed a validated analytical method that combines the electrochemical detection (EC) with ion chromatography (IC). In parallel, we have explored CSF sulfhydrated proteome by top-down proteomics analysis based on matrix-assisted laser desorption/ionization time of flight mass spectrometry (MALDI-TOF MS) in linear mode. In particular, we looked at modifications induced by S-sulfhydration in the cerebral transthyretin (TTR), which are already known to be correlated with some neurological diseases such as MS [56] and AD [57]. Proteomics analysis enabled the investigation of the CSF proteomics profile and the assessment of the alleged correlation between the formation of disulfides and H_2_S levels.

## 2. Results

This work investigates the H_2_S-related CSF profiles in order to reveal putative correlations between H_2_S levels and immuno-inflammatory or degenerative CNS disorders.

### 2.1. Pre-Analytical Stage: Direct Assessment of CSF Sample Quality Prior to H_2_S Investigation

The first preliminary step of the study was to carefully assess the quality of all CSF samples.

This is a mandatory and essential check for proteomics studies on biomarkers. In fact, pre-analytical variables, including temperature, pH, sample storage, and handling, can interfere with the success of the analysis of biofluids, easily leading to phenomena of sample degradation and mainly oxidation [52,58]. In biological systems, proteins are the main non-water components, and therefore represent the main targets of action of radical species such as ROS, reactive nitrogen species (RNS), and also reactive sulfur species (RSS). Redox imbalance leading to proteins oxidation by reactive species is implicated in a wide range of pathological mechanisms leading to neurodegeneration [59]. Hence, it is important to discriminate against degradation and oxidation of the sample due to incorrect pre-analytical procedures and changes induced by pathological mechanisms and oxidative stress.

To begin with, a thorough assessment of all CSFs (7.2–7.5 pH range) was carried out as described by Greco et al. [52].

All samples collected for analysis were stored at −80 °C to avoid oxidation and degradation as much as possible, as suggested for proper proteomics analysis of CSF.

Therefore, to ensure that H_2_S levels were not dependent on other phenomena due to incorrect pre-analytical procedures, the proteomic profiling of all CSFs was checked by direct analysis on MALDI-TOF MS in linear mode.

CSF MALDI-TOF spectra were acquired between 5 and 20 kDa as described in the methods section. Three “guard proteins”, considered as indicators of sample quality state, were assessed. Briefly, each sample was checked for: (i) blood contamination; (ii) Cystatin C degradation; (iii) transthyretin (TTR) degradation.

The whole blood contamination may be due to an alteration in the permeability of the blood CSF barrier or a traumatic lumbar puncture; in both cases a significant addition of blood molecules, including proteins and metabolites, to the CSF may occur, with consequent alteration of the proteomic and metabolomic CSF profile [60].

MALDI spectra of blood-contaminated CSFs usually show characteristic peaks at *m*/*z* = 15,127 Da and 15,868 Da representing the alpha and beta hemoglobin chains, respectively [47]. Moreover, double-charged alpha and beta Hb chains are observed at *m*/*z* = 7564 Da and 7934.5 Da, respectively (Figure S1).

Out of 137 samples, only one CSF with this typical profile was not taken into account. The absence of alpha and beta hemoglobin chains assured us that the H_2_S concentrations measured by our method depend exclusively on the synthesis by nervous system, without H_2_S contaminations by other sources such as blood and peripheral system.

In parallel, CSF MALDI-TOF spectra were zoomed in the 12 kDa region. Samples whose MS spectra showed a peak identified as Cystatin C (CysC) fragment (*m/z* = 12,536 Da), instead of the intact CysC (*m/z* = 13,345 Da), were also discarded. It was ensured that only samples stored correctly were analyzed. The CysC fragment is a well-known ex vivo degradation marker. In human CSF, regardless of health status, a cleavage of CysC, at the N-terminal fragment (*m/z* = 12.5 kDa), is the result of a long-term storage at −20 °C, which would not be present if the same sample had been stored at −80 °C. Indeed, the presence of the *m/z* 12,536 signal is associated with improper storage and handling of samples (Figure S2).

The presence of the signal *m/z* 12,536 is due to improper storage and handling of the samples.

Finally, CSF MALDI-TOF spectra were zoomed in the 13 kDa region and in vitro CSF TTR modifications were evaluated. Human transthyretin (TTR) is a very abundant protein in CSF; its homotetrameric structure contains a free cysteine thiol group in position ten (Cys10) that can be oxidized forming mixed disulfide bridges with a variety of thiol reactive species.

TTR-Cys10-S thiolation is related to different pathological conditions, such as AD [57] and MS [56], so that it is considered a biosensor of the redox neurological status [61]. However, the oxidation of the molecular TTR on Cys10 and the subsequent formation of its isoforms may also depend on pre-analytical variables, such as temperature, pH change, time elapsed, cycles of freezing/thawing [62] and, last but not least, sample aging.

Depending on the temperature increase and ageing of the sample, the monomeric transthyretin (*m/z* 13.76 kDa) shows a tendency to decrease, while, on the contrary, the intensity of the peaks corresponding to its isoforms (*m/z* 13.87, 13.94, and 14.07 kDa), increases. This clearly provides evidence of temperature-dependent TTR oxidation [63] (Figure S3).

Similarly, the increase in pH, due to both the decrease in CO_2_ evaporation and the low buffering capacity of the CSF occurring during sample storage, could lead to mixed disulfide modifications.

CSF TTR as marker of sample oxidation was assessed in the MALDI-TOF spectra, before and throughout the entire study. The increase of these oxidized isoforms was checked in CSF MALDI spectra. This will be further explored below.

Only samples which do not show the oxidized TTR profile were considered.

Following this pre-analytical assay, 134 CSF samples remained for subsequent IC and proteomics investigations.

### 2.2. Chromatographic Method Validation

We then proceeded with the IC detection. The described analytical method has been developed and validated to quantify H_2_S in CSF.

To the best of our knowledge, this is the first study to measure H_2_S on CSF samples related to neurological disorders.

The preparation of CSF samples has been optimized to analyze both free and protein-bound H_2_S fractions. The direct analysis of H_2_S compounds has always been a critical challenge being easily volatile and easy to be oxidized [64,65]. Therefore, following the well-validated protocols for other fluids, some tips have been introduced, as described in the methods section.

First, it should be noted that the amount of sample required for analysis is minimal (30 µL). It is an important advantage considering that CSF sampling is an invasive procedure and cannot be repeated for ethical issues. In addition, some precautions have been taken to measure protein-bound H_2_S. Trichloroacetic acid (TCA) has been used for protein precipitation, as it causes a complete denaturation of proteins and stabilizes the modification of cysteine [66]. Immediate denaturation at low pH prevents further thiol exchange reactions and stabilizes the redox-state within the cellular proteome [67]. With this regard, it should be noted that acid-labile sulfur could also be detected under strongly acidic conditions (pH < 5.4) [14]. Traces of acid-label sulfur may be revealed after the TCA precipitation especially in the supernatant, which is not the matter of the present study.

Similarly, to detect high molecular weight sulfur, the use of tris(2-carboxyethyl)phosphine (TCEP) instead of Dithiothreitol (DTT) was preferred to reduce protein disulfides. TCEP provides some advantages over DTT or β-mercaptoethanol. It is non-volatile, odorless, thiol-free, easily soluble, and a very stable reducing agent. Finally, but above all, TCEP does not give chromatographic interferences with sulfide determination.

Following the recommendations by Giuriati et al. [68] and Xu et al. [69], we chose the electrochemical detection (ED) coupled to the ion chromatographic system as a suitable instrument for H_2_S measurements.

ED offers a rapid, affordable, simple, and real-time tool to measure H_2_S. Particularly, high sensitivity and selectivity, low detection limit, rapid response time, and not requiring the use of additional chemical reagents and derivatives, are the many advantages that make this approach an attractive and reliable method for detecting the concentration of H_2_S in biological samples, as reported for whole blood and mammalian tissues [69,70].

In the amperometric mode that we used, the pH-Ag/AgCl reference electrode is a standard combination pH electrode containing a glass membrane pH half-cell and an Ag/AgCl half-cell.

The combination pH electrode monitors eluent pH.

To overcome the loss of sensitivity, we used disposable silver electrodes easily replaceable that improve the ease of use and robustness of the method. In addition, the use of fresh and degassed mobile phase increases the sensitivity and overcomes the exceeding increase in noise associated with electrolysis.

**Figure 1:**
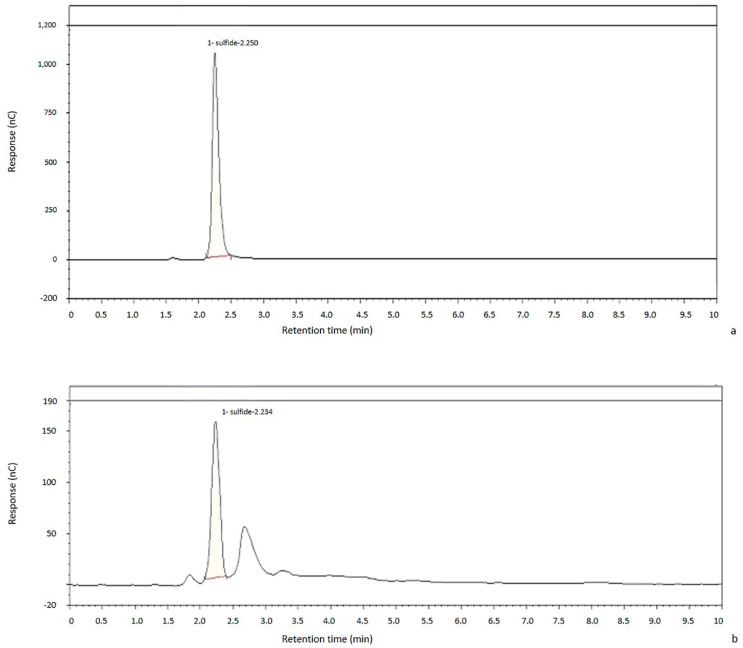
Chromatogram of both a standard (**a**) and a representative cerebrospinal fluid (CSF) (**b**), respectively, have been shown. The sample preparation described does not give any chromatographic interference with sulfide determination. Comparing both chromatograms, sulfide signal shows the exact retention time.

As Figure 1 shows, comparing the CSF sample with NaHS standard, the optimization of CSF sample preparation coupled to the IC-EC system allows an accurate detection of sulfide, without any chromatographic interference (Figure 1).

The present IC method for the determination of sulfide has been optimized and validated internally for linearity, sensitivity, robustness, precision, accuracy, and repeatability.

#### 2.2.1. Linearity

The linearity was assessed by analyzing the detection signals as a function of the analyte concentration, with the aid of a regression line using the calibration curve method. All the correlation coefficients obtained ranged from 99.93 to 99.99% (R^2^ ≥ 0.999).

#### 2.2.2. Sensitivity

The detection limit (limit of detection, LOD) and the quantitation limit (limit of quantitation, LOQ) have been considered. These are two key parameters for determining whether an analyte is present in the sample and for testing the likelihood of false positive and negatives. As described in methods section, LOD and LOQ were calculated based on the standard deviation of the response (SD) and the slope of the calibration curve (S). In this study, LOD, calculated as 3 (SD/S) was 0.009 mg/L; LOQ, calculated as 10 (SD/S), was 0.028 mg/L.

#### 2.2.3. Robustness

To assess the robustness of the method, some analytical parameters were deliberately modified.

As shown in Table 1a,b, respectively, slight changes in the mobile phase (NaOH 70 ± 5 mM) and in flow rate (1.0 ± 0.1 mL/min) did not affect the retention time and peak sulfide area.

#### 2.2.4. Precision and Accuracy

Accuracy and precision were assessed by determining the recovery of known amounts of sulfide in the quality control samples. The intra- and inter-day accuracy and precision studies are shown in Table 2. These results suggest an excellent consistency, precision, and reproducibility of this method.

#### 2.2.5. Recovery

Recovery tests were based on spiking the real sample with known amounts of standard solutions. For recovery experiments, known amounts of NaHS (2, 5, 10 mg/L respectively) were added to CSF samples and then analyzed using the proposed method. Spiked samples were immediately analyzed by IC system.

In spiked samples, the sulfide concentration was calculated by interpolation from the calibration curve and compared with the amount of analytes previously added to the samples. In total, three batches of CSF (pH 7.2, 7.4, and 7.4 respectively) were analyzed four times each. The results of recoveries are shown in Table 3.

The assessment of sample quality by MALDI–TOF MS throughout the study has ensured us that the levels of H_2_S do not depend on the incorrect sample and storage.

### 2.3. H_2_S Concentrations in CSFs

Nowadays, it has been accepted that H_2_S signaling is involved in the regulation of many essential physiological processes in the CNS [5]. Acting as “double-faced Janus” [71], depending on its endogenous concentrations, H_2_S can suppress oxidative stress with positive effects, as it has been shown in animal models of PD [72], and AD [40], on the other hand H_2_S can induce a N-methyl-D-aspartate (NMDA) receptor -independent neuronal death and increase intracellular calcium to toxic concentrations [29].

Taking into account these opposing biological actions, we wondered whether H_2_S levels could vary in a heterogeneous cohort of CSF samples that includes inflammatory and demyelinating autoimmune disorders (ADEM, MS), chronic neurodegenerative diseases (AD, PD), and motor neuron disease (ALS).

Therefore, as described in methods section, each of the all 134 CSF samples was divided separately into two aliquots for the measurements of free H_2_S and bound sulfur -respectively, then both fractions were analyzed by IC system.

First, we evaluated all H_2_S concentrations obtained from the CSF population considering it as a whole. Thus, the measurements obtained from all CSFs were clustered into two groups, free H_2_S and bound sulfur respectively.

**Figure 2:**
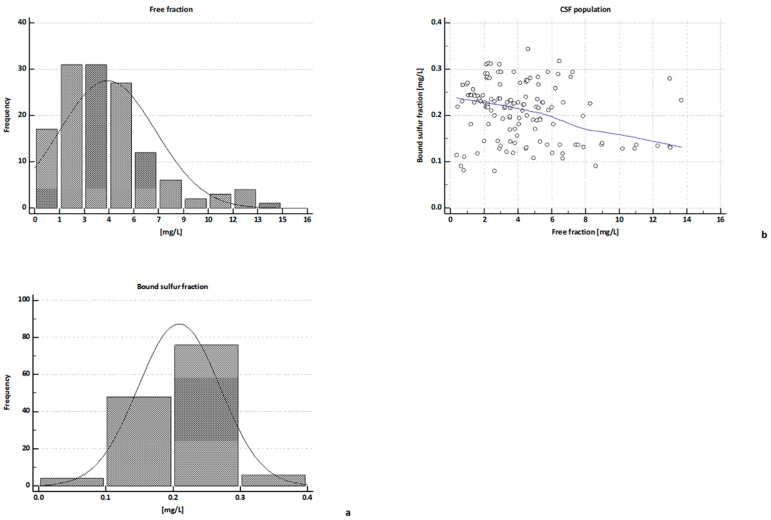
Distribution of H_2_S measurements across the CSF population. All the data collected from IC analysis, were analyzed with MedCalc statistical software; distribution plots, obtained using the non-parametric Kolmogorov–Smirnov (K-S) test, were shown for both fractions free and bound sulfur, respectively, at **top** and **bottom** of the figure (Panel (**a**)). Pearson’s analytical test does not show any correlation comparing the free H_2_S measurements with the bound H_2_S levels (Panel (**b**)).

Figure 2a shows the distribution plots of H_2_S measurements of both groups (free and bound sulfur), respectively.

According to the non-parametric Kolmogorov-Smirnov (K-S) test, the free H_2_S group (Figure 2a, top) does not show a normal distribution (*p* = 0.0007) in the population, with a median value of 3.74 (±0.24) and 5th and 95th percentiles of 0.86 and 10.78 ppm, respectively. It is interesting to note that the minimum and maximum values of H_2_S levels develop over a very wide range from 0.4 ppm to 13.69 ppm.

The K-S distribution of the bound H_2_S concentrations (Figure 2a, bottom) is not normal (*p* = 0.0001), with a median value of 0.22 (±0.005) and 5th and 95th percentiles of 0.11 and 0.29 ppm, respectively. H_2_S levels are included in the range 0.08–0.34 ppm.

Pearson’s analytical test highlights no correlations between bound sulfur and free H_2_S groups (Figure 2b). High concentrations of free H_2_S do not correspond to high levels of protein bound H_2_S as well as low levels of free H_2_S do not correspond to low levels of bound H_2_S.

We can hypothesize that the production of these forms, free and bound sulfur, is independent and does not reflect a simple thermodynamic balance of redox species.

On the basis of this clear heterogeneity, in order to understand what these differences depend on, and if there is any correlation between these two fractions, a more in-depth comparative investigation of H_2_S measurements has been applied among all groups.

The H_2_S concentrations were then assessed on the basis of neurological conditions and compared in all tested clinical groups.

Table 4 shows free and bound sulfur levels measured for all neurological and control groups across the whole population.

As easily notable, the dataset analyzed shows a dynamic variation in H_2_S measurements in such a diverse CSF population.

This is not surprising. This high variability has already been shown in plasma and peripheral blood measurements as reported in several studies that develop methods other than ours [73,74,75].

First, the free H_2_S measurements obtained from each neurological group were compared with the control group. As described in the methods, the control group is composed of patients with no neurodegenerative diseases who mainly suffer from headache, lower back pain, dizziness or vestibular vertigo, and psychiatric disorders.

Significantly different concentrations were shown between the control group (3.48 mg/L ± 1.68) and ALS (6.32 mg/L ± 3.17; *p* = 0.0001), MS (2.07 mg/L ± 0.68; *p* = 0.0001) and PD (5.48 mg/L ± 0.97; *p* = 0.0004) respectively; by contrast, no significant differences were found with ADEM (3.62 mg/L ± 1.4), and AD (4.17 mg/L ± 0.91).

**Figure 3:**
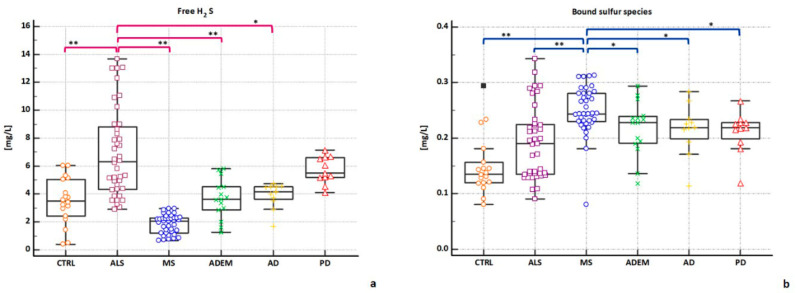
Comparison of free H_2_S and bound sulfur levels in all clinical groups analyzed. Data were analyzed with the Mann–Whitney test. Panel (**a**) shows free H_2_S measurements in all the clinical groups analyzed. Significant differences in free H_2_S levels have been highlighted between ALS group (purple), and the other groups (* *p* < 0.05, ** *p* < 0.0001). Panel (**b**) shows bound sulfur measurements in all the clinical groups analyzed. Significant differences in bound sulfur levels have been highlighted between MS group (blue), and the other groups (* *p* < 0.05, ** *p* < 0.0001). CTRL = control; ALS = Amyotrophic lateral sclerosis; ADEM = Acute Disseminated Encephalomyelitis; AD = Alzheimer’s disease; PD = Parkinson’s disease; MS = Multiple Sclerosis.

Concerning our neurological datasets, despite the variability even within the same clinical group, the average concentration of free H_2_S is higher in ALS (6.32 mg/L ± 3.17) than in other neurological diseases analyzed and controls. The highest significance is shown in comparison with MS (*p* < 0.0001) and controls (*p* < 0.0001), then with ADEM (*p* = 0.0002) and AD (*p* = 0.0018), no significant differences were found with PD group (Figure 3a).

Interestingly, the MS group shows the lowest significant H_2_S free concentrations (2.07 mg/L ± 0.68) in comparison with all the other groups: ADEM (*p* < 0.0001), AD (*p* < 0.0001), PD (*p* < 0.0001), and, as previously stated, with ALS (*p* < 0.0001), and controls (*p* = 0.0001).

Moving on the analysis for bound sulfur fraction, similarly, the measurements obtained from each neurological group were compared with the control group. Significantly low concentrations were shown between the control group (0.13 mg/L ± 0.05) and all the neurological groups analyzed: ALS (0.19 mg/L ± 0.06; *p* = 0.0314); ADEM (0.22 mg/L ± 0.05; *p* = 0.0016); AD (0.22 mg/L ± 0.04; *p* = 0.0075); PD (0.22 mg/L ± 0.04; *p* = 0.007) and MS (0.24 mg/L ± 0.04; *p* < 0.0001).

Among the neurological groups analyzed (0.24 mg/L ± 0.04), the average value of the bound sulfur is significantly higher in MS than in the other neurological diseases: ALS (*p* < 0.0001); ADEM (*p* = 0.015); AD (*p* = 0.009); PD (*p* = 0.0017) (Figure 3b).

Pearson’s analytical test has been applied by comparing bound sulfur and free H_2_S for each group analyzed. No correlation between the two variables was found for CTRL (coefficient correlation r = −0.037; *p* = 0.8812), ALS (r = −0.246; *p* = 0.1282) ADEM (r = 0.1643; *p* = 0.1643) and AD (r = 0.3251; *p* = 0.3251), and PD (r = −0.3128; *p* = 0.3490)

Interestingly, a significant inverse correlation exists for the MS group (coefficient correlation r = −0.6478; *p* < 0.0001): as the concentration of free H_2_S decreases, the bound fraction increases, as well as higher levels of free H_2_S correspond to lower levels of bound sulfur.

**Figure 4:**
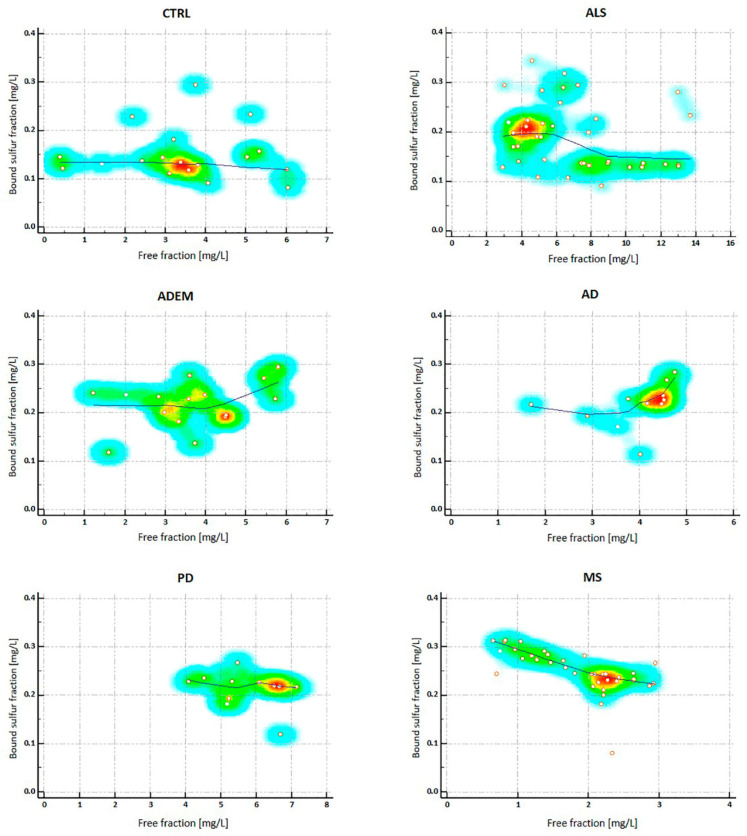
Scatter diagrams on the correlation bound sulfur/free H_2_S in all the clinical groups analyzed. All the data were analyzed with MedCalc statistical software; Pearson’s analytical test have been applied to compare bound sulfur levels with free H_2_S measurements. Free H_2_S and bound sulfur variables define the horizontal axis and the vertical axis respectively. No correlation between the two variables was found for CTRL, ALS, ADEM, AD, and PD. In the last box, a significant inverse correlation has been shown for the MS group (coefficient correlation r = −0.6478; *p* < 0.0001). CTRL = control; ALS = Amyotrophic lateral sclerosis; ADEM = Acute Disseminated Encephalomyelitis; AD = Alzheimer’s disease; PD = Parkinson’s disease; MS = Multiple Sclerosis.

The relationship between the two variables (bound sulfur and free H_2_S) is represented graphically by scatter diagram for each group (Figure 4).

Resuming, taking in consideration the median value, the highest levels of bound sulfur belong to MS group, which shows the lowest values of free H_2_S. Conversely, the highest levels of free H_2_S belong to ALS group, which shows lower values of bound H_2_S with the exception of controls.

### 2.4. MALDI-TOF MS Analysis

In order to further this last issue, we aimed to investigate the CSF proteome linked to S-sulfhydration.

In this perspective, based on the above considerations, we focused on MS and ALS groups that mostly show a different and contrasting profile related to H_2_S levels. Linear MALDI-TOF-MS experiments were performed to explore low protein molecular weight region of CSF, in the range 2–20 kDa, and to obtain high-resolution protein profiling of TTR isoforms related to supposed sulfydration (-SH).

On the basis that the sample’s quality has been previously checked, as described above, we are confident that any modification of TTR could depend on the state of the CNS and the pathological conditions.

As for Cys-10 of TTR, Cysteine residues are extremely sensitive to cellular redox balance. The three oxidation states of sulfur atom of the Cys thiol group can change in response to numerous alterations in the cell redox state. As a result, Cys residues are the main targets of redox modifications which affect the activity of target proteins in determining susceptibility to oxidative damage and neurodegeneration [76].

Our data highlighted that the MS group, with the highest levels of high molecular weight bound sulfur species, shows a characteristic MALDI profile on TTR which is different in comparison to the other neurological groups, and distinguishes MS from the other analyzed diseases.

**Figure 5:**
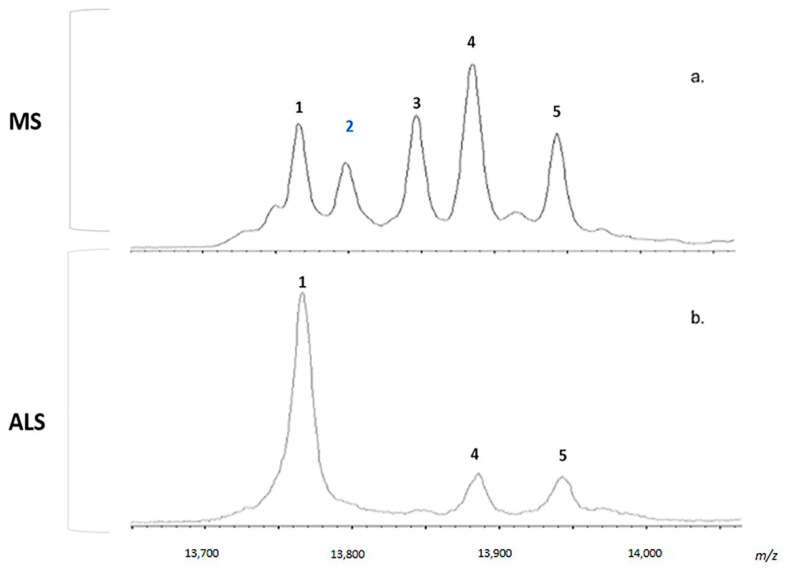
CSF average MALDI-TOF spectra zoomed in the 13 kDa region related to cerebral transthyretin (TTR). Panel (**a**) shows multiple sclerosis (MS) average CSF spectrum, while panel (**b**) shows average spectrum of CSFs with Amyotrophic Lateral Sclerosis (ALS). TTR Free monomeric specie (*m/z* = 13,761 Da, peak 1) and canonical mixed disulfides of monomeric TTR with free thiols with cysteine and cystein-glycine at *m/z* = 13,880 Da (peak 4) and 13,937 Da (peak 5), respectively, are shown for both spectra. MS spectrum also shows mixed disulfides with mass shift of +32 Da (peak 2) and +80 Da (peak 3), with respect to free monomeric specie (*m/z* = 13,761 Da, peak 1), referable to S-sulfhydration, highlighted in blue, (Cys-S-SH) and S-sulfonation (Cys-S-SO3H) of the Cys10 of the protein.

Based on data obtained from IC measurements, we focused mainly on the most appealing comparison between ALS and MS. Particularly, as it has been shown in Figure 5, all the neurological groups, including ALS and MS, show canonical peaks of TTR, which include TTR Free monomeric specie (*m/z* = 13,761 Da, peak 1) and canonical mixed disulfides of monomeric TTR with free thiols with cysteine (*m/z* = 13,880 Da, peak 4) and cystein-glycine (*m/z* = 13,937 Da, peak 5). In addition, as already described by Pieragostino et al. [56], MS TTR shows atypical mixed disulfides with mass shift of +32 Da and +80 Da, compared to free monomeric specie (*m/z* = 13,761 Da). These peaks are generated on the Cys10 of the protein through S-sulfhydration (Cys-S-SH, *m/z* = 13,792 Da, peak 2) and S-sulfonation (Cys-S-SO3H, *m/z* = 13,840 Da, peak 3), respectively, and they are absent in all other CSFs groups, including ALS (Figure 5).

**Figure 6:**
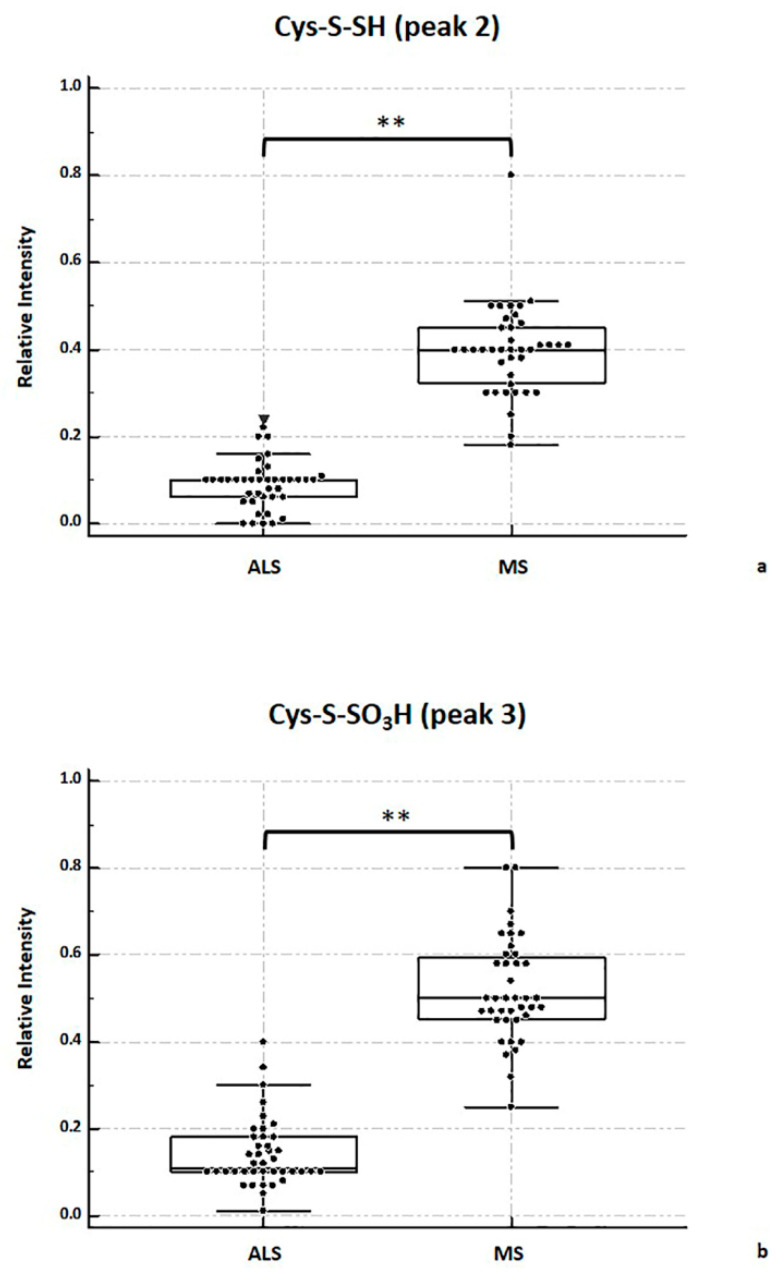
Relative intensity of TTR mixed disulfide isoforms comparing CSF MALDI spectra of ALS and MS groups. Panel (**a**) shows sulfhydrated-TTR isoform (Cys-S-SH, peak 2). Panel (**b**) shows sulfonated TTR isoform Cys-S-SO3H peak 3). Data were analyzed with the Mann–Whitney test; ** indicates a *p* < 0.001.

As Figure 6 shows, the relative intensity of Cys-S-SH peak (peak 2) and Cys-S-SO3H peak (peak 3) has been assessed with respect to the free TTR signal (peak 1) comparing ALS and MS group. Specifically, the sulfhydrated-TTR (peak 2) was significantly higher in MS subjects than in ALS (*p* < 0.001; Figure 6a). Similarly, the relative intensity of the sulfonated TTR was significantly higher in MS compared to ALS (*p* < 0.001; Figure 6b). No significant differences have been shown on TTR monomeric signal (peak 1) and mixed disulfides (peaks 4 and 5) between the two groups.

### 2.5. Considerations

These preliminary results, which require more in-depth investigation of this phenomenon, lead us to wonder whether different mechanisms and pathways related to the synthesis and/or metabolism of H_2_S may be involved in the investigated neuropathologies. In the brain, all the enzymes, CBS, 3-MST, CSE, contribute to the synthesis of H_2_S at different sites.

CBS seems to be a key enzyme in many pathologies related to altered H_2_S concentrations. For example, SOD1^G93A^ mice, mouse model of familiar ALS (fALS), show both higher H_2_S concentrations in brain tissues that WT mice and accumulation of CBS in mitochondria of spinal cord neurons [77]. Thus, similarly, the increase in CSF H_2_S levels, highlighted in our study, could also depend on CBS expression.

In addition, this phenomenon could be linked to the mitochondrial damage associated with ALS.

ALS is a lethal disease characterized by the progressive degeneration of upper and lower motor neurons, especially in the spinal cord. Many factors, such as excitotoxicity and microglia-dependent inflammation, are involved in the ALS cellular damage. However, it is generally accepted that a severe mitochondrial dysfunction associates with the inevitable neuronal death [78].

On the other hand, historically, H_2_S toxicity has been recognized mainly in the inhibition of cytochrome c oxidase (Complex IV) [79,80]. Therefore, in this context, based on our results, it is intriguing to speculate that, in ALS, H_2_S, at such high CSF concentrations, could strengthen a self-amplifying vicious cycle in an already vulnerable motor neuron. Thus, even in motor neurons of ALS patients, as well as in the mouse model, a partial mitochondrial disarrange could be at least partially attributable to the high endogenous concentrations of H_2_S to which they are exposed, possibly caused by the increased expression of CBS.

ALS is not the only disease to be associated with an alteration of H_2_S and CSB. DS, for example, is another neurological disease, whose high concentrations of urinary H_2_S have long been known [49,81,82]. Recently, in fibroblasts from DS individuals, an altered H_2_S concentration has been related to an increase in CBS expression, resulting in the inhibition of mitochondrial Complex IV [83,84].

Investigating an assumed correlation between ALS and DS could be helpful in clarifying which mechanisms might be involved in this alteration.

Several investigations have identified the potential regulatory role of H_2_S through the so-called mechanism of S-sulfhydration (or Persulfidation) [19,85]. This novel PTM, generating a -SSH group, changes the state of a protein critically, and therefore, its physiological action [27,86,87]. Much progress has been made on the correlation between S-sulfhydration and neurodegeneration as reviewed [88].

Clearly the TTR study on MALDI-TOF MS does not give us a comprehensive and complete idea of how much the proteome of ALS, MS, or any other neurological condition, is actually sulfhydrated. We should investigate the whole proteome to achieve such a mapping. However, TTR can give clues of how the sulfhydration mechanism can change the functional activity of a protein. Compared to the physiological condition, the modified TTR decreases its thyroxine-carrying activity, possibly contributing to the demyelination process in MS [56].

The role of thiols/disulfides homeostasis has already been highlighted in MS [89] as well as in AD [90].

MS is generally known as inflammatory and autoimmune disease affecting the CNS. Oxidative stress mainly contributes to the inflammation, and consequent degeneration of myelin and oligodendroglia, leading to axonal damage and, eventually, neuronal death [91]. In this scenario, the thiols/disulfides ratio is crucial in maintaining the redox balance. By acting as an antioxidant buffer, thiols (containing the -SH group) are able to react with ROS to protect cells against the oxidative damage. Alternatively, the disulfide bonds thus generated can be reduced again in the related thiols. Therefore, the balance between antioxidants and oxidants, that is reflected in the thiols/disulfides ratio, allows a correct evaluation of the dynamic redox system of the organism [89]. In MS the shift in favor of disulfides has been especially reported in patients with retrobulbar optic neuritis [92].

Our data on TTR seem to support this evidence; in this context H_2_S and reactive sulfur species could influence the redox imbalance by decreasing the antioxidant action of thiols favoring disulfide formation. This could also explain a high involvement of H_2_S in favor of the formation of sulfur protein compounds (to the disadvantage of the free counterpart), and thus the higher levels of bound sulfur in MS compared to other diseases.

The significant inverse correlation between BSS and free H_2_S, characteristic of the MS group, suggests that the production of these forms, reflects a thermodynamic balance of redox species different from other diseases. We could assume that for diseases such as ALS, the free H_2_S alteration may be due to a disruption of enzymatic production, on the contrary for MS, the thiols/disulfides balance is relevant.

As far we know, studies on the correlation between H_2_S and inflammation show contradictory evidences. Inhibition of endogenous H_2_S may suppress inflammatory responses [93]. Conversely, H_2_S can modulate the inflammatory process by influencing the activities of neutrophils and leukocytes [94].

It is assumed that H_2_S acts close to the sites of its production and that different concentrations of H_2_S at inflammatory sites can induce different cell response such as cellular bioenergetics regulation [95,96]. This can confirm our evidence of a different scenario in ALS motor neurons in comparison to other neuronal sites, such as for MS. A selective vulnerability of motor neurons to H_2_S [97], which is much higher compared to other subtypes of neurons such as the GABAergic neurons [77], has already been shown.

The different concentrations of CSF H_2_S and its forms in the neuropathologies analyzed, could therefore be explained as dependent on the different tropism and the involvement of different neuronal populations, as it occurs, for example, in MS and ALS.

Comparing our dataset on human ALS with studies on both fALS mouse model [45,77] and WT mouse (C57BL/6N) [17], congruent evidence confirms the increase of H_2_S levels in both patients and the ALS animal model compared to controls. However, although it is difficult to make an effective comparison, considering the different models (human vs. mouse) as well as biological samples (CSF vs. tissue), we can infer that the concentration range (µM) seems to be higher in human CSF than in mouse tissues.

This argument could be also applied to PD [98]. An impairment of endogenous H_2_S production appears to be involved in PD. H_2_S decreases in the unilateral striatum injured compared to that in the sham-operated rats in a 6- hydroxydopamine (6-OHDA)-induced PD rat model indicating a possible reduction of the endogenous H_2_S production in the progression of PD [38]. In PD animal models, a dysregulation of H_2_S signaling occurs, contributing especially in the clearance of toxic proteins such as parkin [27], as well as it is evident the neuroprotective beneficial effect of H_2_S donors [99].

All the more so, we believe that accurate detection is also essential for the accurate development of drugs targeted to a particular body district.

To the best of our knowledge there are no data on H_2_S and ADEM. This disease is very rare, and, due to the limited availability of CSF samples, we analysed a few cases. However, it is clear that CSF H_2_S levels in ADEM differ from those found in MS, although they are both autoimmune conditions included in the spectrum of acquired inflammatory demyelinating diseases of the CNS. According to our findings, and notwithstanding that the number of samples was small, in addition to the involvement of other neuronal compartments, probably other different mechanisms, which could also include disease specific etiological factors, are added to and/or interfere with H_2_S production.

Our study supports the hypothesis of an involvement of this gasotrasmitter in neurodegeneration and opens new perspectives to questions that still do not find answers of its precise mechanisms of action in the diseased brain.

Further investigations of free/bound sulfur species ratio in CSF should be carried out in order to ensure clinical validity to the described data. Our results unlock new prospects for investigations that focus even more on the different effects of H_2_S in relation to the neuronal site where it is produced and to its concentrations. Unveiling the molecular basis of its metabolic impairment could help to evaluate whether monitoring its synthesis and metabolism is a valid approach to slow down and/or control the neurodegenerative pathways leading to neuronal death.

## 3. Materials and Methods

### 3.1. Materials

All chemicals used in this study were of analytical grade and purchased from Sigma Aldrich (Sigma-Aldrich S.r.l., Milan, Italy), including H_2_S donor, sodium hydrosulphide hydrate (NaHS) and Trichloroacetic acid (TCA). The reducing agent tris(2-carboxyethyl)phosphine (TCEP) was purchased by Thermo Scientific (Thermo Fisher Scientific, Waltham, MA, USA). MilliQ water was prepared using Milli-Q Gradient A10 system (Millipore, Billerica, MA, USA). Solvents for sample preparation and spotting on MALDI plate were purchased from Fluka (Thermo Fisher Scientific, Waltham, MA, USA).

### 3.2. Sample Collection

The study design followed the ethical standards in conformity with those laid down in the Declaration of Helsinki (World Medical Association, 1997). CSF samples were collected from each subject for standard clinical practice in diagnostic procedures. All the investigations have been performed on the residual sample aliquots (about 40/50 microliters) following the conclusions of all clinical procedures.

In total, 137 CSF samples were collected by a lumbar puncture into sterile polypropylene tubes and centrifuged. The clinical group included: 39 MS; 43 ALS; 15 ADEM; 11 AD; 11 PD and 18 CTRL. Based on extensive diagnostic evaluation, all control patients had no neurodegenerative diseases and no objective clinical, structural (cranial magnetic resonance imaging), laboratory (CSF analysis), or functional (electroencephalography) deficits, suffering predominantly from headache (spondylogenic or nonspecific), lower back pain, dizziness or vestibular vertigo, and psychiatric disorders. Supernatants were transferred to fresh tubes and immediately stored at −80 °C until analysis.

### 3.3. Sample Preparation for MALDI-TOF MS Analysis

Proteins of all 137 CSF samples were extracted and analyzed by MALDI-TOF MS in linear mode according to Pieragostino et al. [56].

Briefly, 20 µL of each sample, acidified with 0.1% TFA, were desalted and concentrated by ZipTip C4 (Millipore, Billerica, MA, USA). Desalted samples were co-crystallized with a saturated solution of sinapinic acid dissolved in 30% acetonitrile, 0.1% trifluoroacetic acid (TFA) on a Ground Steel plate (Bruker-Daltonics, Bremen, Germany), and pre-spotted with a thin layer of a saturated solution of sinapic acid dissolved in 100% ethanol. Mass spectra were acquired with an Ultraflex III MALDI-TOF/TOF spectrometer (Bruker-Daltonics, Bremen, Germany) equipped with SmartBeam laser, working in positive linear mode. The acquisition range was set to 1–20 kDa. External calibration was performed using a mix of peptide calibration standard II (*m/z*: 757.3992, 1046.5418, 1296.6848, 1347.7354, 1619.8223, 1758.9326, 2093.0862, 2465.1983, 3147.4710; Bruker-Daltonics, Bremen, Germany) and protein calibration standard II (*m/z*: 5734.51, 6180.99, 8476.65, 8565.76, 12,360.97, 16,952.30; Bruker-Daltonics, Bremen, Germany).

Data processing and interpretation were performed by Flex Analysis 3.3 (Bruker-Daltonics, Bremen, Germany) and ClinProTools 3.3 (Bruker-Daltonics, Bremen, Germany) softwares.

This protocol on MALDI-TOF MS has been applied for both the CSF quality assessment and the TTR investigation.

### 3.4. Sample Preparation for H_2_S Measurements (Free and Bound Sulfur Fractions)

Sample preparation has been performed following the protocols by Bailey et al. [100] and Giuriati et al. [68] for measuring H_2_S in biological matrices with some modifications. Briefly, after the assessment of sample quality, all CSF samples (*n* = 134, 7.2–7.5 pH range) were divided in two aliquots to measure free and bound sulfur fractions, respectively. Each aliquot (30 µL) was thawed on ice and mixed with MilliQ water (ratio 1:5 *v*/*v*). The solution was then mixed and incubated on ice for 10 min.

Preliminary stage of protein precipitation was necessary to measure protein bound sulfur fraction. Proteins were precipitated by adding TCA 12% (ratio 1:1 *v*/*v*) and the mixture was vortexed. After 1 h of incubation at −20 °C, the samples were spun at 15,000 g for 30 min at 4 °C, and the supernatant was removed. Then, protein pellets were suspended with phosphate buffer saline (PBS, 100 mM, pH = 7.4). A solution of tris(2-carboxyethyl)phosphine (TCEP, final concentration 1mM, Thermo Fisher Scientific, Waltham, MA, USA) immediately prepared, as recommended to ensure TCEP stability, and incubated for 30 min. Samples were then cooled to reduce the disulfide binding. Removal of excess TCEP is not required.

All aliquots (free and bound sulfur) from all CSFs were analyzed in triplicate by ion chromatography (IC). The H_2_S concentration was calculated against a calibration curve of NaHS (0.05–20 mg/L), as described above. Bound sulfur levels were normalized over protein concentration (µg/µL). Protein concentration was determined using the Bradford assay.

### 3.5. Ion Chromatography (IC) for H_2_S Measurements

#### 3.5.1. Chromatographic Condition: Instrumentation and Procedures

Chromatographic analyses were carried out using the metal-free ion chromatograph Model ICS3000 system (Thermo Fisher Scientific, Waltham, MA, USA). This model is equipped with an electrochemical detector (ED) that includes a Dual Pump; an AS50 autosampler, an EG Eluent Generator; a DC detector/Chromatography with an Ag electrochemical detector, stainless steel and Ag/AgCl, KCl (sat) electrodes used as working, counter and reference, respectively.

Sulfide separation was carried out using the IonPac™ AS15 Hydroxide-Selective Anion-Exchange Column 5 µm (3 × 150 mm, Thermo Fisher Scientific, Waltham, MA, USA) protected by a IonPac™ AG15 Guard Columns 5 µm (3 × 30 mm, Thermo Fisher Scientific, Waltham, MA, USA). Column was maintained at room temperature to avoid potential fouling.

A NaOH 75 mM solution, prepared through the NaOH Eluent Generator (Thermo Fisher Scientific, Waltham, MA, USA), was the mobile phase solution; the flow rate was 0.5 mL/min. All measurements were performed using an isocratic eluent delivery in triplicates. The synthetic samples were degassed for at least 10 min and the eluent solutions were continuously degassed with helium. The volume injected was 25 µL.

The sulfide detection was performed with a disposable silver working electrode and an Ag/AgCl reference electrode.

Chromatographic and Pulsed Amperometric Detection (PAD) conditions are summarized in Table 5.

#### 3.5.2. Standard Solutions

For each sample, H_2_S level was determined using the NaHS calibration curve just prepared and analyzed on the same batch. Standard has been dissolved in phosphate buffer saline (100 mM, pH 7.4). Standard calibration levels have been set at 0.05, 0.1, 1, 5, 10, and 20 mg/L.

#### 3.5.3. Linearity and Range

Calibration curves were constructed from standard NaHS solutions at a concentration range of 0.05–20 mg/L, plotting the peak area of the NaHS standard (*y*-axis) against the corresponding sulfide concentrations (*x*-axis). Slope regression, intercept, and correlation parameters were calculated with linear regression analysis.

In order to assess the sensitivity of the method, the detection limit (limit of detection, LOD) and the quantitation limit (limit of quantitation, LOQ) were calculated in the samples based on the standard deviation of response (SD) and the slope of the calibration curve (S), according to the formulas: LOD = 3 (SD/S) and LOQ = 10 (SD/S).

#### 3.5.4. Intra and Inter-Day Precision

To assess the accuracy and precision of this method samples quality control was assessed for intra-day evaluation. In addition, this analysis was carried out for three consecutive days to determine the inter-day variability.

#### 3.5.5. Recovery

A recovery study was conducted by comparing peak areas obtained from samples spiked with known amount of NaHS (at low, medium and high levels) and those obtained from the untreated samples.

### 3.6. Data and Statistical Analysis

All instrument control, data acquisition, and data analysis for H_2_S measurements were performed using the Chromeleon software (v6.8, Dionex, Thermo Fisher Scientific, Waltham, MA, USA). H_2_S concentration was calculated against a calibration curve of NaHS (0.05–20 mg/L). For each CSF sample, protein bound sulfur levels were normalized to the protein concentration (µg/µL).

MedCalc software (MedCalc Software, Mariakerke, Belgium) was used for statistical analysis. Non-parametric Kolmogorov–Smirnov (K-S) test and distribution plots were used to analyze H_2_S measurements across the population.

Wilcoxon Mann–Whitney test was performed for comparing clinical groups. Person’s analytical tests were performed for correlation analyses.

The values of *p* < 0.05 were considered significant. The 95% of confidence interval was assumed for each test.

Mass spectrometry data were analyzed as described above.

## 4. Conclusions

In order to elucidate the role of H_2_S in the brain, we developed and validated an analytical chromatographic method to measure its forms, free and bound sulfur, in CSF samples from a heterogeneous population of patients, which includes several neurological conditions. Based on the obtained measurements, a wide and heterogeneous distribution of CSF H_2_S concentrations was highlighted. In such an extreme variability, among the neurological groups analyzed, ALS and MS clusterize as the two groups with the highest concentrations of free H_2_S and the highest concentration of bound sulfur, respectively. In addition, based on our proteomics speculations, a different proteomics profiling characterizes these diseases; in particular, H_2_S-related S-sulfydration is detected only on the TTR of patients with MS. What does it depend on? We are aware that our investigation has some limitations to draw concrete conclusions. The biochemical mechanisms underlying this effect are currently not fully understood and they would require additional functional examinations which are not part of the current study.

We believe that the strength of this research is the application of a reliable chromatographic analytical method for measuring H_2_S in a biofluid as valuable as CSF. This study is the first clinical report on H_2_S concentrations in CSF from patients with neurodegenerative diseases.

The detection of endogenous CSF H_2_S concentration, integrated with the evaluation of clinical features, opens new perspectives in view of the potential clinical relevance of H_2_S for the prognosis and monitoring of neurodegenerative diseases. It could also provide useful information for drawing potential clinical treatment strategies and for the development of new H_2_S-based therapy.

## Figures and Tables

**Table 1:** Assessment of robustness modifying mobile phase (**a**) and flow rate (**b**). Each measurement was performed on quadruplicate; the mean and the Relative standard deviation (RSD%) have been evaluated for both concentration (mg/L) and retention time (RT), respectively.

(**a**)												
**Sulfide**	**NaOH 70 mM**				**NaOH 75 mM**				**NaOH 80 mM**			
[mg/L]	[mg/L]	RSD%	R T	RSD%	[mg/L]	RSD%	R T	RSD%	[mg/L]	RSD%	R T	RSD%
2	1.98	3.9	2.28	3.9	2.1	10	2.17	0.31	1.95	6.4	2.08	6.4
5	5.6	6.3	2.28	6.3	5.2	6.8	2.15	1.02	5.2	3.5	2.08	3.49
10	9.4	4.8	2.28	4.8	10.2	2.4	2.17	0.23	9.7	7.0	2.11	7.10
(**b**)												
**Sulfide**	**0.9 mL/min**				**1.0 mL/min**				**1.1 mL/min**			
[mg/L]	[mg/L]	RSD%	R T	RSD%	[mg/L]	RSD%	R T	RSD%	[mg/L]	RSD%	R T	RSD%
2	2.19	8.0	2.33	0.52	2.1	10	2.17	0.31	2.04	5.1	2.02	0.19
5	5.1	6.0	2.34	0.41	5.2	6.8	2.15	1.02	4.79	7.9	2.02	0.5
10	10.7	8.7	2.36	2.71	10.2	2.4	2.17	0.23	10.7	6.4	2.01	0.39

**Table 2:** Assessment of intra- and inter-day accuracy and precision, measured by relative standard deviation RSD%, and relative error RE%.

Sulfide	Intra-Day		Inter-Day	
(mg/L)	RSD%	RE%	RSD%	RE%
2	5.07	2.14	7.65	2.48
5	7.89	5.24	7.70	3.89
10	6.37	2.40	7.72	1.47

**Table 3:** Assessment of recovery by the analysis of three batches of CSF, each was analyzed four times.

Spiked Concentration	Batch 1		Batch 2		Batch 3	
[mg/L]	Mean%	RSD %	Mean%	RSD %	Mean%	RSD %
2	98.9	0.6	104.7	0.7	100.3	0.7
5	100.6	2.1	99.4	3.2	98.4	1.5
10	97.9	5.4	94.8	2.9	96.7	3.1

**Table 4:** H_2_S measurements across the CSF population. Data were analyzed with the Mann–Whitney test; values (mg/L) are presented as median, with ranges in parentheses.

	CSF Group (*n* = 134)	CSF Groups					
		CTRL (*n* = 18)	ALS (*n* = 40)	MS (*n* = 39)	ADEM (*n* = 15)	AD (*n* = 11)	PD (*n* = 11)
free H_2_S	3.74	3.48 **	6.32	2.07 **	3.62 *	4.17 *	5.48
	(13.69–0.39)	(6.04–0.39)	(13.69–2.92)	(2.95–0.66)	(5.8–1.23)	(4.75–1.71)	(7.16–4.09)
Bound Sufur	0.21	0.13 ^§§^	0.19 ^§§^	0.24	0.22 ^§^	0.22 ^§^	0.22 ^§^
	(0.34–0.08)	(0.29–0.08)	(0.34–0.09)	(0.31–0.08)	(0.29–0.12)	(0.28–0.11)	(0.27–0.12)

Significant differences in free H_2_S levels have been highlighted between ALS group, and the other groups (* *p* < 0.05, ** *p* < 0.0001). Significant differences in bound sulfur levels have been highlighted between MS group, and the other groups (^§^ *p* < 0.05, ^§§^ *p* < 0.0001). CTRL = control; ALS = Amyotrophic lateral sclerosis; ADEM = Acute Disseminated Encephalomyelitis; AD = Alzheimer’s disease; PD = Parkinson’s disease; MS = Multiple Sclerosis.

**Table 5:** Chromatographic and detection conditions.

**Column**	IonPac™ AS15 Hydroxide Selective Anion-Exchange Column 5 µm (3 × 150 mm, Thermo Fisher Scientific) + IonPac™ AG15 Guard Columns 5 µm (3 × 30 mm, Thermo Fisher Scientific)		
**Eluent**	NaOH 75 mM solution prepared by NaOH Eluent Generator (Thermo Fisher Scientific)		
**Injection volume**	25 μL		
**Detection**	disposable silver electrode		
**PAD sequence**	Time	Potential	Integration
	0	−0.1	Begin End
	0.2	−0.1	
	0.3	−0.1	
	0.31	0.1	
	0.4	0.1	

Technical parameters have been schematized both for chromatographic (column, guard column, eluent, injection volume) and detection analysis (electrode and Pulsed Amperometric Detection condition-PAD-).

## Data Availability

The data collected in this study are contained within the article and supplementary material.

## Supplementary Materials

The following are available online at https://www.mdpi.com/2218-1989/11/3/152/s1, Figure S1: CSF mass spectra with blood contamination, Figure S2: CSF mass spectra with Cystatic C degradation, Figure S3: CSF mass spectra with TTR oxidation.
