# Supplementary material for: Investigating Different Forms of Hydrogen Sulfide in Cerebrospinal Fluid of Various Neurological Disorders
Source: Metabolites. 2021 Mar 8;11(3):152. doi: 10.3390/metabo11030152 (PMC7998212; doi:10.3390/metabo11030152)

Figure S1: CSF mass spectra with blood contamination.
CSF MALDI-TOF MS spectra acquired between 5-20 kDa, using sinapinic acid as matrix. Panel A shows a typical spectrum with blood contamination containing hemoglobin (Hb) chains compared to one without contamination (Panel B).

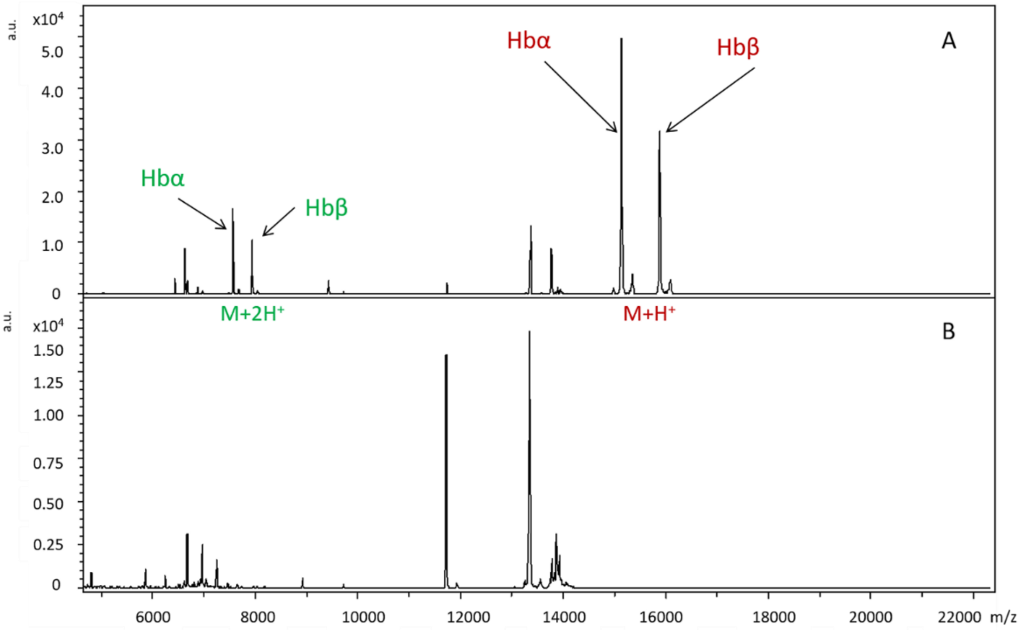

The range between 15–17 kDa and 7–9 kDa presents signals matching with Hb chains with one (red) and two (green) charges. CSF Hb chains alpha and beta are observed at m/z = 15 127 Da and 15 868 Da (red), respectively. Double-charged Hb chains alpha and beta are observed at m/z =7564 Da and 7934.5 Da (green), respectively.

Figure S2: **CSF mass spectra with Cystatic C degradation**.
CSF MALDI-TOF spectra acquired between 5-20 kDa, using sinapinic acid as matrix and zoomed into the 12 kDa region.
The figure shows the cystatin C (CysC) signals obtained from CSF samples after storage at -80°C (panel A) and -20°C (panel B) respectively.
In panel B a cleavage of CysC, at the N-terminal fragment (m/z= 12.5 kDa), is the result of long-term storage at -20°C, which is not present for the CSF sample stored at -80°C.

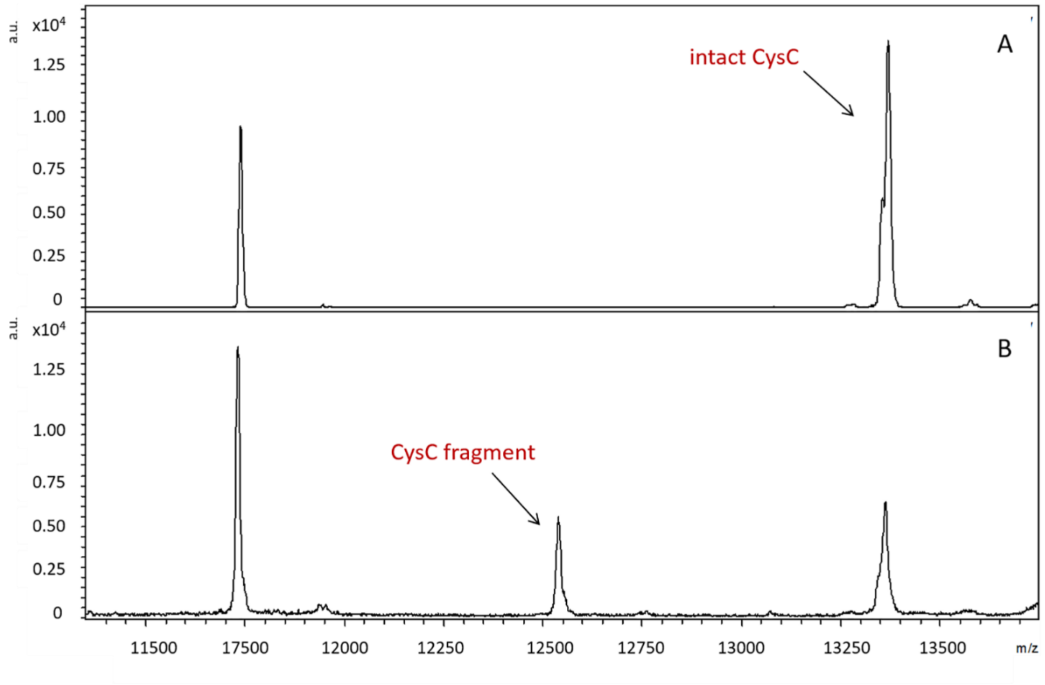

Figure S3: **CSF mass spectra with TTR oxidation**.

CSF MALDI-TOF spectra acquired between 5-20 kDa, using sinapinic acid as matrix and zoomed in the 13 kDa region.

The figure shows the transthyretin (TTR) signals obtained from fresh CSF (panel A) and aged (panel B) samples respectively. Panel A shows typical spectra of TTR with the free monomeric form at m/z= 13761 Da; panel B provides evidence of TTR oxidation by showing a decrease in monomeric TRR instead of an increase in the intensity of the peaks corresponding to its mixed disulfide isoforms.

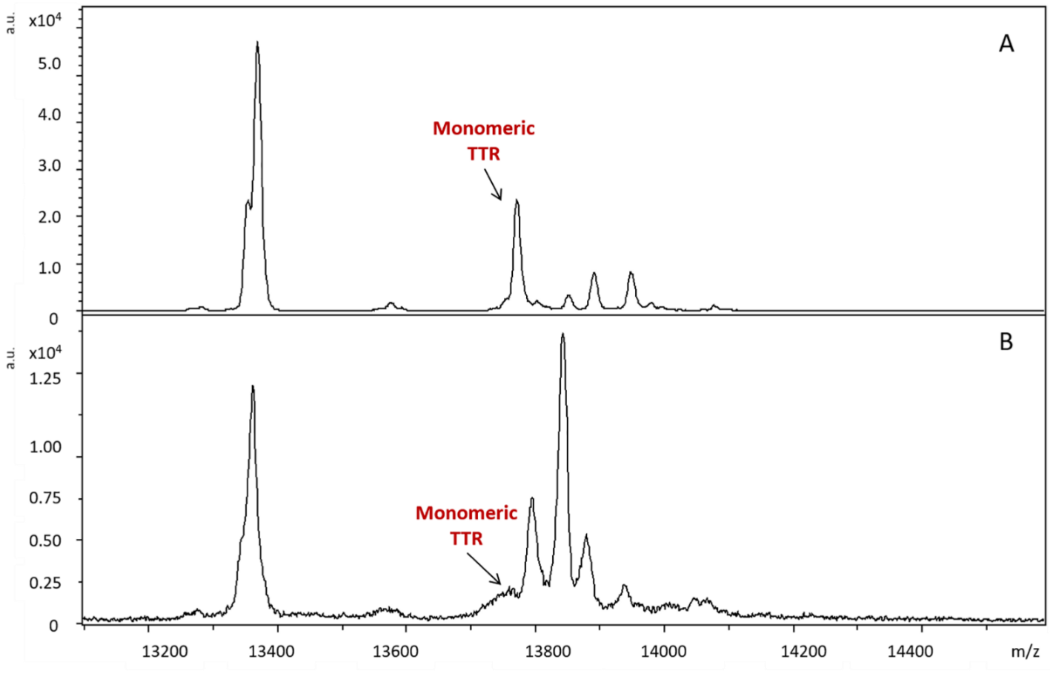

Supplement: Supplementary file 1 [file metabolites-11-00152-s001.pdf]
